# Comparison of fecal microbiota of SPF and non-SPF Beagle dogs

**DOI:** 10.3389/fvets.2023.1021371

**Published:** 2023-02-07

**Authors:** Cuilian Yu, Zhongkun Guo, Zhan Lei, Xiaolong Mao, Shumin Chen, Kezhou Wang

**Affiliations:** ^1^School of Laboratory Animal and Shandong Laboratory Animal Center, Shandong First Medical University and Shandong Academy of Medical Sciences, Jinan, China; ^2^Shandong Provincial Center for Animal Disease Control and Prevention (Zoonoses Surveillance Center of Shandong Province), Jinan, China

**Keywords:** Beagle dogs, fecal microbiota, SPF, alpha diversity, environmental adaptability

## Abstract

Microbial colonization of animal intestine impacts host metabolism and immunity. The study was aimed to investigate the diversity of the intestinal microflora in specific pathogen free (SPF) and non-SPF Beagle dogs of different ages by direct sequencing analysis of the 16S rRNA gene. Stool samples were collected from four non-SPF and four SPF healthy Beagle dogs. From a total of 792 analyzed Operation taxonomic units, four predominant bacterial phyla were identified: Firmicutes (75.23%), Actinobacteria (10.98%), Bacteroidetes (9.33%), and Proteobacteria (4.13%). At the genus level, Streptococcus, Lactobacillus, and Bifidobacterium were dominated. Among which, Alloprevotella, Prevotella_9, and Faecalibacterium were presented exclusively in non-SPF beagles, with potentially anti-inflammatory capability, which could protect non-SPF beagles from complex microbial environment. The number and diversity of intestinal flora for non-SPF Beagle dogs were the highest at birth and gradually decreased with growth, whereas the results for the SPF beagle samples were the opposite, with the number and diversity of intestinal microbiota gradually increases as beagles grow. In a nutshell, the microbial complexity of the rearing environment can enrich the gut microbiota of beagles, many of which are anti-inflammatory microbiota with the potential to increase the adaptability of the animal to the environment. However, the gut microbiota of SPF beagles was more sensitive to environmental changes than that of non-SPF beagles. This study is of great significance for understanding the bionomics of intestinal microflora in non-SPF and SPF beagles, improving the experimental accuracy in scientific research.

## 1. Introduction

Experimental animals played a key role in scientific and medical research. With the development of modern life science, the quality requirements of experimental animals are increasing. Conventional experimental animals have complex microbial states, which cannot meet the requirements of scientific research and production. Therefore, SPF experimental animals have become more important research work. Beagle as a standard non-rodent experimental animal has been widely applied due to its submissive behavior, medium size, long life span, and consistent genetic delivery. Therefore, it is very necessary to understand the biological characteristics of SPF beagles in research work.

Gut microbiota, which is closely associated with host nutrition, metabolism, and immunity, acts as a “second genome” for modulating the health phenotype of the superorganism host (1, 2). As an internalized “microbial organ,” the gut microbiota provides many functions that are not encoded by the host genome but shown to be essential for animal health, such as short-chain fatty acid (SCFA) production, vitamin synthesis, and immune system regulation (3–5). Such health-relevant functions may be considered ecosystem services provided by the gut microbiota to the host (6). Gut microbiota is also involved in animal's health and diseases. The gut microbial composition is related with dog's metabolism and immunity in many processes (7–9). In comparison, based on the dog gut microbiome gene catalog presented by Coelho et al., the dog microbiome is closer to the human microbiome than the microbiome of either pigs or mice (7). Therefore, the findings in beagle dogs may be predictive of human microbiome results. However, there are limited information about the fecal microbiota of SPF and non-SPF Beagle dogs.

High-throughput sequencing plays an increasingly important role in biological sample for studying the population structure, microbiome diversity, and evolution of the bacterial flora of humans and animals (1, 10, 11). This study analyzed the 16S rRNA gene sequences of intestinal flora in SPF and non-SPF beagles by high throughput sequencing and elucidated the development and differences of intestinal flora in beagles. The results showed that the microbiota was more abundant in the stool samples from non-SPF beagles, which may reduce the sensitivity of non-SPF beagles to environmental change.

## 2. Materials and methods

### 2.1. Animals and management

Four non-SPF and four SPF newborn Beagle dogs from Qingdao Bolong Experimental Animal Co., Ltd were enrolled into this study. Throughout the study, none of the dogs received drugs, such as gastrointestinal disease drugs, antibiotics, or diabetes drugs, that could affect their gut microbiota, nor did they receive additional nutritional supplements. SPF beagles are bred in pairs in sterilized isolators and fed artificially sterilized breast milk until they are 1 month old, then gradually weaned on a dry diet. After weaning, they were fed sterilized maintenance commercial extrusion (dry-type) diet with free access to sterilized water. The isolator maintains a positive pressure of 118–127 Pa, humidity of 40–70% and 30 air changes per hour. The temperature inside the isolator is 34–35 degrees for the first week, and then decreases by 2 degrees every week until it reaches 24–26 degrees. Non-SPF beagles were raised in pairs in spacious indoor enclosure, kept at the same temperature and humidity as the SPF beagles' isolation unit, breastfed naturally until 1 month of age, then gradually weaned, after which they were fed the same sterilized maintenance commercial extruded (dry-type) diet as SPF dogs and were given free water. All dogs gain behavioral enrichment through interaction with each other, playtime with their caregivers, and access to toys.

### 2.2. Sample collection

Fresh stool samples from newborn, 1 and 3-month-old non-SPF Beagle dogs or 1 and 3-month-old SPF Beagle dogs were collected immediately after spontaneous defecation, immediately frozen at −80 °C without any additives or pretreatment, and sent to Shanghai Majorbio Bio-pharm Technology Co., Ltd. for the 16S gene library construction, quantification, and sequencing. Twelve stool samples for non-SPF Beagle dogs were divided into three groups, named F1, F3 and F5, which were collected from newborn, 1 and 3-month-old non-SPF Beagle dogs, respectively. Eight stool samples for SFP Beagle dogs were divided into two groups, named F2 and F4, which were collected from 1 and 3-month-old SPF Beagle dogs, respectively.

### 2.3. Sequence processing and bioinformatic analysis

After sequencing, paired-end reads were assembled using FLASH software (v1.2.11) based on overlap. MOTHUR (v 1.30.2) software is used for quality control and filtering of assembled sequences. Uparse (v 7.0.1090) was used for OTU cluster analysis, and Usearch (v 7.0) was used for taxonomic analysis. Taxonomy based on the 16S rRNA gene sequence was assessed using the Ribosomal Database Project (RDP) classifier (v 2.11) against the Silva database at a confidence level of 0.7. Microbial diversity in the individual stool samples was estimated using rarefaction analysis. Alpha diversity and beta diversity indexes were calculated using MOTHUR. PICRUSt (v 1.1.0) was used to predict the KEGG and COG functions of 16S sequences. The alpha diversity for each sample was measured by OTUs using Sobs, Shannon, Simpson, ACE, Chao, and Coverage indexes. Statistical analyses were performed using Student's *t*-test or analysis of variance (ANOVA) on at least three independent replicates. *P* values of < 0.05 were considered statistically significant for each test (^*^P < 0.05; ^**^P < 0.01; ^***^P < 0.001).

## 3. Results

### 3.1. Sequencing results and acquisition of operational taxonomic units

Stool samples of non-SPF (*n* = 12) and SPF (*n* = 8) Beagle dogs were assessed using high-throughput sequencing. After removing low-quality sequences and non-target regions, the number of cleaned sequences were 914,442 reads, approximately 45,722 reads per sample. Among them, 547,960 filtered sequences were obtained from the non-SPF Beagle dogs (180,541 for the F1 group, 1874,96 for the F3 group and 179,923 for the F5 group) and 366,482 filtered sequences were from SPF Beagle dogs (185,520 for the F2 group and 180,962 for the F4 group).

All sequences were classified for OTU analysis according to the similarity threshold setting (0.97). All the stool samples showed 792 OTUs, with an average of 423 bp high-quality sequences (the shortest sequence was 209 bp; the longest sequence was 522 bp). For the non-SPF Beagle dogs, 726 OTUs, with an average of 422 bp (the shortest sequence was 209 bp; the longest sequence was 522 bp), and for the SPF Beagle dogs, 347 OTUs, with an average of 426 bp (the shortest sequence was 216 bp; the longest sequence was 496 bp) were acquired by sequencing.

The number of OTUs for the non-SPF and SPF Beagle dogs fit a normal distribution. For the non-SPF Beagle dogs, there was a significant difference in the number of OTUs (*P* = 0.007) between F1 (newborn) and F3 (1-month-old) groups; but no significant difference (*P* = 0.103) between F3 (1-month-old) and F5 (3-month-old) groups. For the SPF Beagle dogs, there was a significant difference in the number of OTUs (*P* = 0.003) between F2 (1-month-old) and F4 (3-month-old) groups.

**Figure 1:**
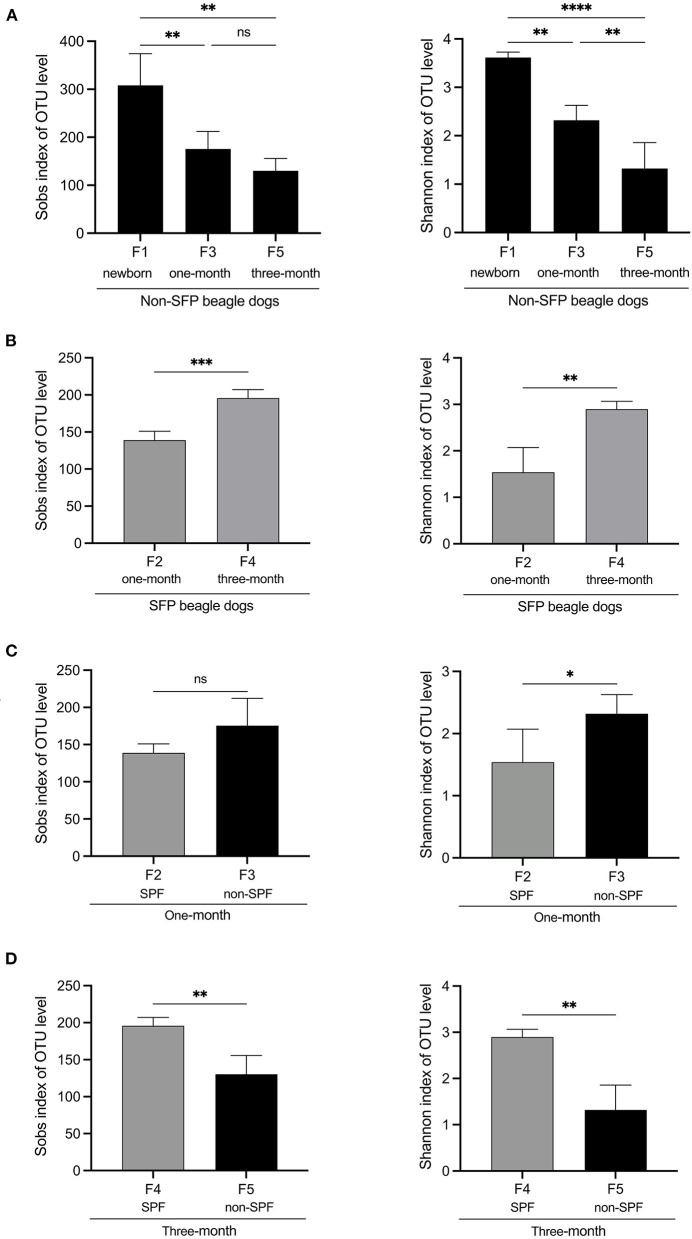
Alpha diversity comparisons of fecal microbiota of non-SPF and SPF beagles. **(A)** Sobs and Shannon species richness indices for the samples of F1 (newborn[[Inline Image]]), F3 (1-month-old) and F5 (3-month-old) groups in non-SPF Beagle dogs. **(B)** Sobs and Shannon species richness indices for the samples of F2 (1-month-old) and F4 (3-month-old) groups in SPF Beagle dogs. **(C)** Sobs and Shannon species richness indices for 1-month-old non-SPF (F3) and SPF (F2) Beagle dogs. **(D)** Sobs and Shannon species richness indices for 3-month-old non-SPF (F5) and SPF (F4) Beagle dogs. The data represent the means ± the SD of four independent samples. Statistical analyses were performed by ANOVA, using GraphPad Prism software (*P < 0.05; **P < 0.01; ***P < 0.001; ****P < 0.0001; “ns” indicates no statistical significance).

Alpha diversity is an ecological measurement of how many taxonomic groups are presenting within each sample and whether the abundance of these groups is evenly distributed. The sequencing data was analyzed by alpha diversity analysis, including the coverage of each sample library, Shannon index, Sobs index, Simpson index, ACE index, and Chao 1 index (Table 1). Compared to the newborn samples for non-SPF Beagle dogs, 1 and 3-month samples were significantly reduced in alpha diversity, using either the sobs index or the Shannon entropy metric, especially the 3-month samples (Figure 1A). However, the alpha diversity of intestinal microbiota for SPF Beagles dogs increased with growth (Figure 1B). The alpha diversity of intestinal microbiota was compared between non-SPF and SPF beagles at the same age (Figures 1C, D). For 1-month-old beagles, the alpha diversity of intestinal microbiota of non-SPF beagles was slightly higher (Figure 1C), whereas the results for 3-month beagles (Figure 1D) were opposite.

**Table 1:** Number of sequences, number of OTUs and alpha diversity indexes retrieved.

**Variable**		**Non-SPF**		**SPF**	
	**Newborn**	**1-month**	**3-month**	**1-month**	**3-month**
Sequence number	45,135.25 ± 2,685.599	46,874 ± 5,392.285	44,980.75 ± 4,592.759	46,380 ± 4,691.262	45,240.5 ± 4,483.114
OTUs	302.5 ± 55.505	163.5 ± 23.553	127.3 ± 22.665	136.5 ± 12.298	189.8 ± 14.906
sobs	308 ± 66.322	175.5 ± 36.711	130.25 ± 25.539	139 ± 11.916	196 ± 11.165
Shannon	3.618 ± 0.109	2.321 ± 0.306	1.323 ± 0.537	1.5411 ± 0.53	2.8995 ± 0.165
Simpson	0.06 ± 0.008	0.195 ± 0.066	0.434 ± 0.243	0.458 ± 0.208	0.109 ± 0.024
ACE	332.78 ± 74.981	360.15 ± 41.657	242.97 ± 44.576	266.36 ± 17.811	285.15 ± 41.094
Chao 1	330.36 ± 70.674	297.45 ± 32.269	204.32 ± 21.388	220.39 ± 25.567	280.81 ± 59.939
Coverage	0.999 ± 0.001	0.999 ± 0.001	0.999 ± 0.001	0.999 ± 0.001	0.999 ± 0.001

According to the rarefaction curves of the five groups (data not shown), as the number of sequences increased, the curve tended to be flat, indicating that the sequencing of each sample was sufficient to reflect the species diversity in the sample.

These results indicated that the number and diversity of intestinal flora for non-SPF Beagle dogs were the highest at birth and gradually decreased with growth. In SPF beagles, in contrast, the number and diversity of intestinal microbiota gradually increases as beagles grow.

### 3.2. Comparison of microbiota at the phylum and genus level

The results of microbiota analysis at the phylum level for the five groups of stool samples demonstrated that of the 4 phyla identified, the content of *Firmicutes* (75.23%) was the highest, followed by *Actinobacteria* (10.98%), *Bacteroidetes* (9.33%), and *Proteobacteria* (4.13%).

**Figure 2:**
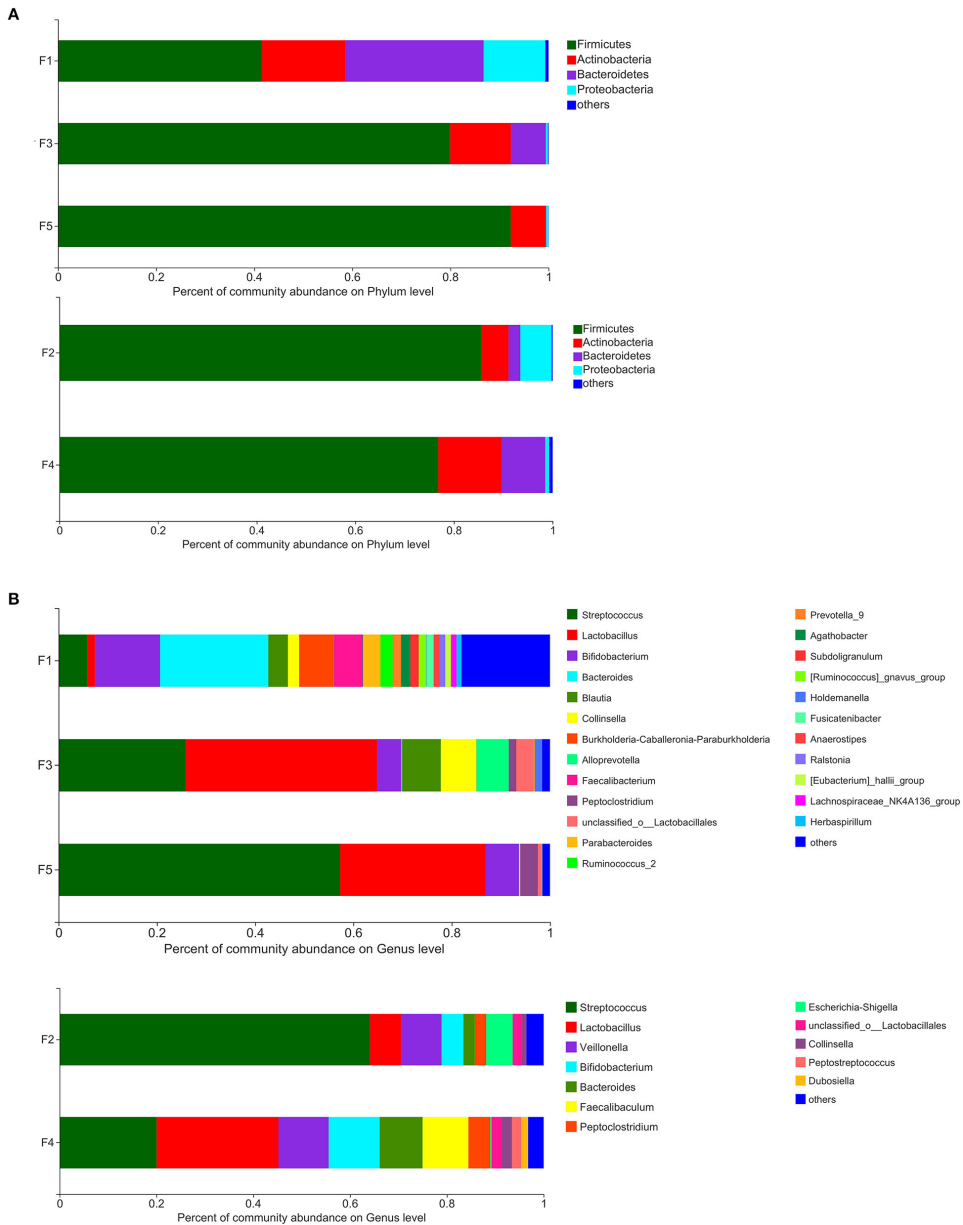
Percent of community abundance of fecal microbiota of non-SPF and SPF beagles. Colored stacked bars indicate the identity of the dominant taxa. **(A)** Percent of community abundance on phylum level for the samples of F1 (newborn), F3 (1-month-old) and F5 (3-month-old) groups in non-SPF Beagle dogs and the samples of F2 (1-month-old) and F4 (3-month-old) groups in SPF Beagle dogs. **(B)** Percent of community abundance on genus level for the samples of F1 (newborn), F3 (1-month-old) and F5 (3-month-old) groups in non-SPF Beagle dogs and the samples of F2 (1-month-old) and F4 (3-month-old) groups in SPF Beagle dogs.

The intestinal microbiota of the five groups of Beagle dogs differed at the phylum level (Figure 2A). Although *Firmicutes* was the dominant taxa in all the five groups, the percentage of *Firmicutes* was different. In non-SPF beagles, with the dog's growth, the proportion of *Firmicutes* in the intestinal flora gradually increased, to 92.27% at the age of 3 months. At the same time, the proportion of *Actinobacteria, Bacteroidetes*, and *Proteobacteria* decreased gradually. In SPF dogs, the proportion of *Firmicutes* and *Proteobacteria* declined slightly with age, while the proportion of *Actinobacteria* and *Bacteroidetes* increased. Different from the previous reported that *Fusobacteria* ranks the third in the intestinal microbiota of healthy beagles (12), the predominant phyla of intestinal microbiota in the non-SPF and SPF beagles in our study did not contain *Fusobacteria*.

Based on the analysis at the genus level, the microbiota was more abundant in the stool samples from non-SPF beagles, which consists of 21 main genera (content percentage >0.01%), whereas the microbiota in the stool samples from SPF beagles is composed of 12 main genera (Figure 2B). On the top of the list for all beagles were *Streptococcus, Lactobacillus*, and *Bifidobacterium*. It is notable that there are many bacterial genera presented exclusively in non-SPF beagles, such as *Burkholderia-Caballeronia-Paraburkholderia, Alloprevotella, Faecalibacterium, Prevotella_9, [Ruminococcus]_gnavus_group, Blautia*, and so on. While *Veillonella, Escherichia-Shigella, Peptostreptococcus*, and *Faecalibaculum* were presented exclusively in SPF beagles. In general, the fecal flora of the non-SPF beagles contains more genera than that of the SPF beagles, and they each contain a few unique bacterial genera. These results suggested that the breeding environment may have some influence on the species of the fecal flora in beagles, and microbial environment complexity could enrich the microbiome.

### 3.3. Functional analysis

**Figure 3:**
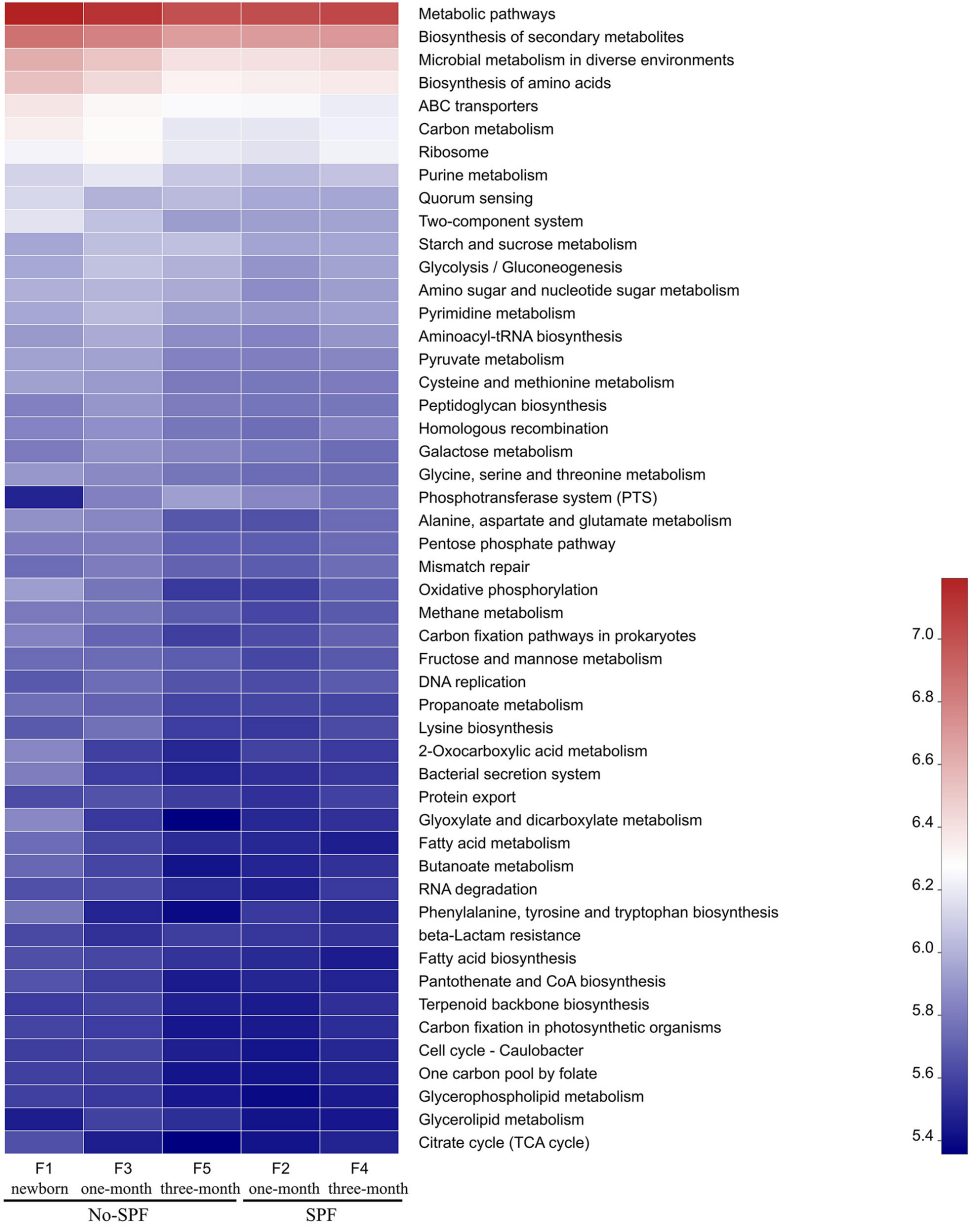
The functional distribution of intestinal microbiota in non-SPF and SPF beagles. Abscissa is the group name, ordinate is the function name, and color gradient is used to show the changes of different function abundance in the group, and legend is the value represented by color gradient.

PICRUSt software was used to compare the species composition information obtained from 16S sequencing data, and to predict the composition of functional genes in the samples, to analyze the functional differences between different samples. KEGG pathway analysis predicted many basic metabolic function genes, among which the abundant genes were Metabolic pathways, Biosynthesis of secondary metabolites, Microbial metabolism in diverse environments, Biosynthesis of amino acids, etc. In general, the functional distribution of intestinal microbiota in non-SPF and SPF beagles was similar (Figure 3). For non-SPF beagles, the functions of the intestinal flora tend to be concentrated with growth, and most functions taking a small proportion at the age of 3 months. Data from 1 and 3-month-old SPF beagles show that the function of the intestinal flora becomes more complex with growth. This trend is consistent with our previous results on species abundance of intestinal flora.

By comparing the functional distribution of intestinal microbiota of 1-month old non-SPF and SPF Beagle dogs, we found that the abundance of pathways related to Microbial metabolism in diverse environments and Biosynthesis of amino acids was higher in the non-SPF group than in the SPF group. This may be related to the high levels of *Lactobacillus, Blautia* and *Prevotella* in the non-SPF Beagle intestinal microflora (Figure 2B). It has been documented that *Lactobacillus* was involved in carbohydrate and protein metabolism (1, 13, 14). *Blautia* was a major producer of short-chain fatty acids, especially acetic acid (15). *Prevotella* was involved in *de novo* synthesis of amino acids and the synthesis of several B vitamins (16, 17). At 3 months of age, the abundance of Microbial metabolism in diverse environments and Biosynthesis of amino acids related pathways in the non-SPF group was lower than that in the SPF group, which might be the effect of *Bifidobacterium, Bacteroides, unclassified_o_Lactobacillales, Faecalibaculum* and *Pepto streptococcus* with higher abundance in the intestinal microbiota of SPF Beagle dogs (Figure 2B). *Bifidobacterium* and *Bacteroides* are known to have bile salt hydrolase enzyme-coding genes and are also participated in carbohydrate metabolism, *Bacteroides, Lactobacillus*, and *Streptococcus* were identified microbial species for their role in proteolysis or amino acid production (13, 14, 16). In addition, *Bacteroidetes* was the major group involved in the synthesis of vitamin B and conjugated linoleic acid, and *Faecalibaculum* was a major producer of SCFA (1, 18). The abundant gut flora provides a platform for metabolic interaction between host and microbiome.

## 4. Discussion

In this study, the fecal microbiota of non-SPF and SPF Beagle dogs, and different ages were characterized to understand the biological characteristics of intestinal microbiota in different rearing environment.

The alpha diversity was compared by the Coverage index, Sobs index, Shannon index, Simpson index, ACE index, and Chao 1 index for the fecal microbiota of the non-SPF and SPF beagles (Table 1). The results showed no significant differences in both richness and biodiversity between the non-SPF and SPF samples. For 1-month-old beagles, the fecal microbial diversity of non-SPF beagles was slightly higher, whereas the results for 3-month-old beagles were the opposite. What is clear, however, is that there is a significant difference in the trend of fecal microbial diversity between non-SPF and SPF beagles as they grow. In non-SPF beagles, the Sobs and Shannon indices decrease gradually with growth and are highest at birth. In SPF beagles, on the other hand, the diversity of gut microbes increased with age (Figure 1).

There was no significant difference in the composition of fecal microbes between the two groups of beagles at the phylum levels, with predominantly *Firmicutes, Bacteroidete, Proteobacteria* and *Actinobacteria*. *Firmicutes* were the main flora of the two groups of beagles, which was consistent with many studies on animal intestinal flora (19), but the abundance trends of the fecal microbes were different. With the growth of non-SPF beagles, the proportion of *Firmicutes* increased gradually, and reached 92.27% by the age of 3 months. In SPF beagles, the proportion of *Firmicutes* decreased slightly with growth. This was contrary to the trend of fecal flora diversity (Figure 2A). Most of the literature reported that the predominant microbial phyla for maintaining gastrointestinal health includes *Firmicutes, Bacteroidetes, Fusobacteria, Proteobacteria* and *Actinobacteria* in dog's gut (12, 14). In contrast, *Fusobacteria* was not the dominant phylum in the beagle's intestinal flora in present study, which may be caused by animal breeds, ages, diets, living environments or experimental methods. We believe that the most important reason may be the age of the beagle dogs. Most of the studies used beagle dogs as adults, but this study was conducted until the beagle dogs were 3 months old, focusing on the impact of living environment on the intestinal flora construction process of the puppies. The predominant phyla in healthy human intestinal flora are *Firmicutes, Bacteroidetes, Actinobacteria* and *Proteobacteria*, suggesting that the intestinal microflora of young dogs may be more similar to that of humans than that of adult dogs.

Based on the analysis at the genus level, *Streptococcus, Lactobacillus* and *Bifidobacterium* were the main fecal flora of both non-SPF and SPF beagle dogs. The three dominant genera are bacteria genera that widely exist in the digestive tract of animals, some strains of *Lactobacillus* and *Bifidobacterium* can also act as probiotics to improve the distribution of intestinal flora and antagonize the colonization of harmful bacteria, so as to protect intestinal health (20, 21).

Moreover, *Burkholderia-Caballeronia-Paraburkholderia, Alloprevotella, Faecalibacterium, Prevotella_9, [Ruminococcus]_gnavus_group* and *Blautia* were present exclusively in non-SPF beagles. *Alloprevotella, Prevotella_9* and *Faecalibacterium* can produce SCFAs (22, 23), who strengthen barrier functions through mechanisms such as G-protein-coupled receptor-mediated sensitization of the intestinal epithelial cells (IECs) inflammasomes, reducing IEC oxygen concentrations and induction of hypoxia-induced factor. Furthermore, SCFAs exert anti-inflammatory and tolerogenic effects on immune cells (24, 25). *Blautia* is a relevantly abundant taxonomic group present in the microbiome of mammalian gastrointestinal tracts, which plays certain roles in host metabolism, inflammation and biotransformation, and has potential probiotic properties (15). We speculated that among the environmental factors separating the two beagles (diet, sanitation, and hygiene) the presence of *Burkholderia-Caballeronia-Paraburkholderia, Prevotella_9, Faecalibacterium* and *Blautia* could be a consequence of complexity of microbial environment, protecting them from inflammations and non-infectious intestinal diseases.

*Veillonella* (26) and *Escherichia-Shigella* (27) peculiar to SPF beagles were opportunistic pathogens, which do not cause healthy animal infection in SPF breeding conditions with uncontaminated air and foods, but there is the possibility of disease when the environment changes. It can be inferred that the environmental adaptability and resistance of SPF beagles were weaker than those of non-SPF beagles.

*Bacteroidetes*, also the SCFA producing bacteria (28, 29), were found in both non-SPF and SPF beagles with a higher proportion, indicating the importance of maintaining a potentially anti-inflammatory microbiota.

KEGG pathway analysis predicted many basic metabolic function genes of intestinal flora. The abundance of functional genes in intestinal flora of non-SPF Beagle dogs decreased with growth, while that of SPF beagle dogs increased with growth. At 1 month of age, the abundance of functional genes in intestinal microbiota of non-SPF Beagles was mostly higher than that of SPF beagles, which may be related to the high concentration of *Lactobacillus, Blautia* and *Prevotella* in intestinal microbiota. However, at 3 months of age, the abundance of many functional genes in SPF beagles was higher than that in non-SPF beagles, which may be due to the higher content of *Bifidobacterium, Bacteroides, unclassified_o_Lactobacillales, Faecalibaculum* and *Pepto streptococcus* in the intestinal flora of SPF Beagles. By participating in the metabolism of carbohydrates, proteins, SCFA, bile salts and vitamins in the gut, these bacteria help the host decompose food and obtain nutrients, improve the utilization of proteins, carbohydrates and vitamins.

The experimental animals used in this study were newborn beagles in general environment and SPF environment. In order to obtain newborn puppies with similar genetic background of the same strain in the same breeding farm at the same time, only 4 animals were obtained in each group, 2 females and 2 males. The number of experimental animals is relatively small but representative. In addition, due to the limitation of experimental conditions, we failed to collect fecal samples from newborn SPF beagles, which is a pity of this study.

This study laid the foundation for the study of SPF and non-SPF Beagle fecal microbiota. By comparing the composition and diversity of the fecal microbiota of non-SPF and SPF beagles, and different ages, we found that living and dietary exposure to the large variety environmental microbes could increase the potentially beneficial bacterial genomes, enriching the microbiome, enhancing the anti-inflammatory ability of intestinal flora. This may be the result of beagles that adapt to the complexity of microbial environment, indicating a role of microbiota in protecting beagles from pathogens. The gut microbes of SPF beagles were more sensitive to environmental changes than that of the non-SPF beagles, which may lead to a weaker environmental adaptability of SPF beagles. It is of great significance for understanding the bionomics of intestinal microflora in non-SPF and SPF beagles, improving the experimental accuracy in scientific research.

## Data availability statement

The data presented in the study are deposited in the NCBI repository, SRP402573, accession number PRJNA890408.

## Ethics statement

The animal study was reviewed and approved by Laboratory Animal Ethics Committee of Shandong Laboratory Animal Center.

## Author contributions

CY retrieved literatures, provided tables, figures, and wrote the manuscript. ZG retrieved literatures and provide methods and techniques. KW proposed the topic and provided outline. ZL revised the manuscript. XM and SC reviewed the final manuscript. All authors contributed to the article and approved the submitted version.

## References

[1] Adak A Khan MR. An insight into gut microbiota and its functionalities. Cell Mol Life Sci. (2019) 76:473–93. 10.1007/s00018-018-2943-4 30317530 PMC11105460

[2] Jia W Li H Zhao L Nicholson JK. Gut microbiota: a potential new territory for drug targeting. Nat Rev Drug Discov. (2008) 7:123–9. 10.1038/nrd2505 18239669

[3] Flint HJ Scott KP Louis P Duncan SH. The role of the gut microbiota in nutrition and health. Nat Rev Gastroenterol Hepatol. (2012) 9:577–89. 10.1038/nrgastro.2012.156 22945443

[4] Rooks MG Garrett WS. Gut microbiota, metabolites and host immunity. Nat Rev Immunol. (2016) 16:341–52. 10.1038/nri.2016.42 27231050 PMC5541232

[5] Bilotta AJ Cong Y. Gut microbiota metabolite regulation of host defenses at mucosal surfaces: implication in precision medicine. Precis Clin Med. (2019) 2:110–9. 10.1093/pcmedi/pbz008 31281735 PMC6598739

[6] Costello EK Stagaman K Dethlefsen L Bohannan BJM Relman DA. The application of ecological theory toward an understanding of the human microbiome. Science. (2012) 336:1255–62. 10.1126/science.1224203 22674335 PMC4208626

[7] Coelho LP Kultima JR Costea PI Fournier C Pan Y Czarnecki-Maulden G. Similarity of the dog and human gut microbiomes in gene content and response to diet. Microbiome. (2018) 6:72. 10.1186/s40168-018-0450-3 29669589 PMC5907387

[8] Li Q Lauber CL Czarnecki-Maulden G Pan Y Hannah SS. Effects of the dietary protein and carbohydrate ratio on gut microbiomes in dogs of different body conditions. MBio. (2017) 8:e01703–16. 10.1128/mBio.01703-16 28119466 PMC5263242

[9] Hullar MAJ Lampe JW Torok-Storb BJ Harkey MA. The canine gut microbiome is associated with higher risk of gastric dilatation-volvulus and high risk genetic variants of the immune system. PLoS ONE. (2018) 13:e0197686. 10.1371/journal.pone.0197686 29889838 PMC5995382

[10] Guan Y Zhang H Gao X Shang S Wu X Chen J. Comparison of the bacterial communities in feces from wild versus housed sables (*Martes zibellina*) by high-throughput sequence analysis of the bacterial 16S rRNA gene. AMB Express. (2016) 6:98. 10.1186/s13568-016-0254-4 27734418 PMC5061668

[11] Metzker ML. Emerging technologies in DNA sequencing. Genome Res. (2005) 15:1767–76. 10.1101/gr.3770505 16339375

[12] Chun JL Ji SY Lee SD Lee YK Kim B Kim KH. Difference of gut microbiota composition based on the body condition scores in dogs. J Anim Sci Technol. (2020) 62:239–46. 10.5187/jast.2020.62.2.239 32292931 PMC7142278

[13] Yadav M Verma MK Chauhan NS. A review of metabolic potential of human gut microbiome in human nutrition. Arch Microbiol. (2018) 200:203–17. 10.1007/s00203-017-1459-x 29188341

[14] Pilla R Suchodolski JS. The role of the canine gut microbiome and metabolome in health and gastrointestinal disease. Front Vet Sci. (2019) 6:498. 10.3389/fvets.2019.00498 31993446 PMC6971114

[15] Liu X Mao B Gu J Wu J Cui S Wang G. Blautia-a new functional genus with potential probiotic properties? Gut Microbes. (2021) 13:1–21. 10.1080/19490976.2021.1875796 33525961 PMC7872077

[16] Lin R Liu W Piao M Zhu H. A review of the relationship between the gut microbiota and amino acid metabolism. Amino Acids. (2017) 49:2083–90. 10.1007/s00726-017-2493-3 28932911

[17] Uebanso T Shimohata T Mawatari K Takahashi A. Functional roles of B-vitamins in the gut and gut microbiome. Mol Nutr Food Res. (2020) 64:e2000426. 10.1002/mnfr.202000426 32761878

[18] Jandhyala SM Talukdar R Subramanyam C Vuyyuru H Sasikala M Nageshwar Reddy D. Role of the normal gut microbiota. World J Gastroenterol. (2015) 21:8787–803. 10.3748/wjg.v21.i29.8787 26269668 PMC4528021

[19] Biddle A Stewart L Blanchard J Leschine S. Untangling the genetic basis of fibrolytic specialization by *Lachnospiraceae* and *Ruminococcaceae* in diverse gut communities. Diversity. (2013) 5:627–40. 10.3390/d5030627

[20] Guandalini S. Probiotics for prevention and treatment of diarrhea. J Clin Gastroenterol. (2011) 45(Suppl):S149–53. 10.1097/MCG.0b013e3182257e98 21992955

[21] Kok CR Hutkins R. Yogurt and other fermented foods as sources of health-promoting bacteria. Nutr Rev. (2018) 76:4–15. 10.1093/nutrit/nuy056 30452699

[22] Flint HJ Bayer EA Rincon MT Lamed R White BA. Polysaccharide utilization by gut bacteria: potential for new insights from genomic analysis. Nat Rev Microbiol. (2008) 6:121–31. 10.1038/nrmicro1817 18180751

[23] Sokol H Pigneur B Watterlot L Lakhdari O Bermúdez-Humarán LG Gratadoux J-J. *Faecalibacterium prausnitzii* is an anti-inflammatory commensal bacterium identified by gut microbiota analysis of Crohn disease patients. Proc Natl Acad Sci U S A. (2008) 105:16731–6. 10.1073/pnas.0804812105 18936492 PMC2575488

[24] Tilg H Adolph TE Gerner RR Moschen AR. The intestinal microbiota in colorectal cancer. Cancer Cell. (2018) 33:954–64. 10.1016/j.ccell.2018.03.004 29657127

[25] Scheppach W Weiler F. The butyrate story: old wine in new bottles? Curr Opin Clin Nutr Metab Care. (2004) 7:563–7. 10.1097/00075197-200409000-00009 15295277

[26] Poppleton DI Duchateau M Hourdel V Matondo M Flechsler J Klingl A. Outer membrane proteome of *Veillonella parvula*: a *Diderm firmicute* of the human microbiome. Front Microbiol. (2017) 8:1215. 10.3389/fmicb.2017.01215 28713344 PMC5491611

[27] Sims GE Kim S-H. Whole-genome phylogeny of *Escherichia coli*/*Shigella* group by feature frequency profiles (FFPs). Proc Natl Acad Sci U S A. (2011) 108:8329–34. 10.1073/pnas.1105168108 21536867 PMC3100984

[28] Xu Y Zhu Y Li X Sun B. Dynamic balancing of intestinal short-chain fatty acids: the crucial role of bacterial metabolism. Trends Food Sci Technol. (2020) 100:118–30. 10.1016/j.tifs.2020.02.026

[29] Pilla R Suchodolski JS. The gut microbiome of dogs and cats, and the influence of diet. Vet Clin North Am Small Anim Pract. (2021) 51:605–21. 10.1016/j.cvsm.2021.01.002 33653538

